# The oncogenic role of the cerebral endothelial cell adhesion molecule (CERCAM) in bladder cancer cells in vitro and in vivo

**DOI:** 10.1002/cam4.3955

**Published:** 2021-06-08

**Authors:** Yali Zuo, Xiaoliang Xu, Minfeng Chen, Lin Qi

**Affiliations:** ^1^ Deportment of urology Xiangya Hospital Central South University Changsha China; ^2^ Department of Pediatric Surgery Binzhou Medical University Hospital Binzhou China

**Keywords:** bladder cancer, cell invasion, cell proliferation, cerebral endothelial cell adhesion molecule (CERCAM), the PI3K/AKT signaling

## Abstract

Bladder cancer is a menace to global health worldwide due to its high recurrence rate and its progression to invasive muscular complications. Cell adhesion molecules play an intricate role in cancer migration, growth, and invasion. Therefore, through bioinformatics analysis, it was found that the higher cerebral endothelial cell adhesion molecule (CERCAM) predicted lower chance in bladder cancer patient survival; subsequently, in vitro and in vivo investigations were performed to evaluate the specific effects of CERCAM on bladder cancer cell phenotypes and tumor growth in mice model. The PCR‐based analysis revealed an aberrant upregulation of CERCAM in bladder carcinoma tissues and cells when compared with normal controls. In vitro, functional experiments such as MTT, EdU, and Transwell assays showed that CERCAM overexpression markedly enhanced bladder cancer cell viability, DNA synthesis, and cell invasion. In contrast, CERCAM silencing suppressed bladder cancer cell viability, DNA synthesis, and cell invasion. CERCAM overexpression significantly increased PCNA, Vimentin, Twist, and N‐cadherin proteins but decreased E‐cadherin and cleaved‐caspase3, whereas CERCAM silencing exerted opposite effects on these markers. In vivo, subcutaneous implant model experiments in nude mice showed that CERCAM silencing suppressed the growth of subcutaneously implanted tumors. CERCAM altered the phosphorylation process of AKT. The PI3K inhibitor LY294002 treatment manifested similar effects as CERCAM silencing on bladder cancer cell behaviors and partially impaired the promotive functions of CERCAM overexpression upon the capacity of bladder cancer cells to proliferate and invade. When taken together, the cell adhesion molecule CERCAM is overexpressed in bladder cancer tissues. In vitro, CERCAM overexpression significantly promoted bladder cancer cell viability, DNA synthesis, and cell invasion and alters the cleaved‐caspase3, E‐cadherin, and N‐cadherin expression pattern; in vivo, CERCAM silencing suppressed tumor growth in nude mice. The PI3K/AKT signaling is suspected of interfering participate in the functions of CERCAM in bladder carcinoma.

## Graphical abstract

In conclusion, cell adhesion molecule CERCAM is overexpressed in bladder cancer tissues. In vitro, CERCAM overexpression not only significantly promotes bladder cancer cell viability, DNA synthesis, and cell invasion, but also alters E‐cadherin and N‐cadherin expression pattern; in vivo, CERCAM silencing suppressed tumor growth in nude mice. The PI3K/AKT signaling might participate in the functions of CERCAM in bladder cancer.

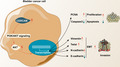

## 1 INTRODUCTION

Bladder cancer is one of the leading causes of cancer‐related deaths worldwide due to its high rates of morbidity and fatality.^1^ This can be attributed to the fact that its prognosis and treatment regimen have remained unchanged in over two decades.^2^ Bladder cancer is still a debilitating disease, often fatal, and one of the costliest cancers to treat and manage. The 5‐year survival rate is still unsatisfactory due to its high recurrence rate and the progress to muscle‐invasive subtypes.^3^, ^4^, ^5^ An in‐depth comprehension of the underlying mechanism of the metastasis of primary bladder carcinoma is essential to fulfil to research for novel treatment regimens.

An essential clinical step in bladder cancer is the progress to or the presentation of muscle‐invasive subtype. Cell‐to‐cell adhesion molecules cause cells to agglutinate at a specific location, break linkages and migrate in a directed way.^6^, ^7^ Classical cadherins, in which E‐, P‐, and N‐cadherin are members, are the best described and understood subset of cadherins and are shown to regulate cell‐cell adhesion in the way as mentioned above.^7^ Bladder cancer is typically characterized by the reduced expression of the cell adhesion molecule E‐cadherin with grade and stage progression, and elevated expression of N‐ or P‐cadherin within muscle‐invasive tumors. These alterations in cell adhesion molecules, known as cadherin switching, have a lasting influence on the cells' phenotypes and behaviors.^8^, ^9^, ^10^, ^11^ In addition to facilitating cell migration to tissues with a consistent cadherin expression pattern (such as vascular endothelium) and away from the epithelial compartment,^9^, ^10^, ^12^ the switching might also alter intracellular signaling to optimize the growth and the invasive traits of the cells.^9^, ^12^ The critical roles of the cell adhesion molecules and the cadherin switching within tumor migration, growth, and invasive ability were taken into consideration. This triggered the research for previously un‐studied cell adhesion molecules related to cell survival, migration, growth, and invasion.

Recently studies involving whole‐genome and sequencing have successfully identified the genes and genetic pathways of the key drivers of bladder carcinoma. Generally, multiple signaling pathways, including the PI3K/AKT, MAPK, Wnt, etc.,^13^ are altered at the initial stage or during bladder cancer development. The PI3K/AKT is one of the most extensively studied targets in bladder cancer treatment. The Cancer Genome Atlas has recently identified the alteration of the RTK/RAS/PI3K/AKT/mTOR signaling pathway in 72% of bladder cancer cases.^14^ The PI3K signaling pathway is frequently in a dysregulated state in tumors and may be inhibited by various available small molecule inhibitors, therefore becoming an essential anticancer target. Notably, epithelial cell adhesion molecule (EpCAM) was associated with prostate cancer metastasis and chemo‐ or radio‐resistance through the PI3K/AKT signaling.^15^ It is thus speculated that the PI3K/AKT signaling might also have a role to play in cell adhesion molecules in bladder carcinoma.

Herein, online data mining using data from The Cancer Genome Atlas Urothelial Bladder Carcinoma (TCGA‐BLCA) data collection was carried out, in view of identifying cell adhesion molecules related to the survival of patients suffering from bladder cancer. Among cell adhesion molecules that were significantly associated with patients' survival, cerebral endothelial cell adhesion molecule (CERCAM) yielded the lowest p‐value. Therefore, CERCAM was chosen for further experiments. CERCAM expression was confirmed within tissues and cells. Furthermore, CERCAM overexpression or silencing was conceived in bladder cancer cells, and the effects of CERCAM on the behaviors of bladder cancer cells were analyzed. A subcutaneous implant model was conducted in nude mice, and the in vivo effects of CERCAM silencing on tumor growth were examined and recorded. Finally, CERCAM‐overexpressing bladder cancer cells were treated or non‐treated with the PI3K inhibitor LY294002. The dynamic effects of CERCAM overexpression and LY294002 upon bladder carcinoma cell proliferation and invasion were examined. Altogether, the present study aims at identifying the potential cell adhesion molecule that modulates bladder cancer cells' capacity to proliferate and invade, subsequently uncovering its potential mechanism.

## 2 MATERIALS AND METHODS

### 2.1 Tissue sample collection

Twelve paired bladder cancer tissues and adjacent non‐cancerous tissues were harvested during tumor biopsies. Initial queries of bladder cancer were established after physical examinations and ultrasound imaging by an urologist. The diagnosis was subsequently confirmed through cystoscopy and tumor tissue analysis by a pathologist. All the samples were immediately fixed in formalin or stored at −80°C following surgical resection waiting further investigation. None of the patients had undergone any antitumor therapy before histological diagnosis. Also, clinical sampling was performed with the approval of the Medical Institutional Ethics Committee of Xiangya Hospital, Central South University. Informed consent was signed and obtained from each patient enrolled. The clinical characters were listed in Table S1.

### 2.2 qRT‐PCR (real‐time quantitative PCR)

Through the use of TRIZOL™ (Catalog number: 15596026; Invitrogen), total RNA from target tissues or cells was isolated. cDNA was synthesized using an oligo ethylene glycol‐based transcript first‐strand cDNA synthesis kit (Product No.: 04896866001; Roche Diagnostics, Basel, Switzerland). For cDNA synthesis, 500 ng of total RNA was used, and the total RNA was diluted with RNAse‐free H_2_O, and the total RNA's final concentration was adjusted to 5 ng/mol. RT‐qPCR detection was performed using a Power SYBR Green PCR master mix (Catalog number: 4367659; Life Technologies) in an ABI Prism 7900HT instrument (Applied Biosystems). The target gene's relative expression level was calculated using the 2^−ΔΔCt^ method. The β‐actin level level (for mRNA expression) was used as an internal reference. The primers were listed in Table 1.

**TABLE 1:** The primer sequence

	F	R
qRT‐PCR CERCAM	GAGCCCAGGTTCTACCCAGAT	GCAGAGTCTGATTGTTGGTCA
qRT‐PCR β‐actin	TTCCAGCCTTCCTTCCTGGG	TTGCGCTCAGGAGGAGCAAT
lv‐CERCAM	ctaccggactcagatctcgagATGCGCGCTGCCCGCGCC	gtaccgtcgactgcagaattcCTAGAGCTCATCTCGGGGCTG
Lv‐sh‐NC	GATCCGTGAGCCTGCATGGGATGGATCTCGAGATCCATCCCATGCAGGCTCACTTTTTG	AATTCAAAAAGTGAGCCTGCATGGGATGGATCTCGAGATCCATCCCATGCAGGCTCACG
Lv‐sh1‐CERCAM	GATCCGCTTTGAGCTAGGCTAGAGATCTCGAGATCTCTAGCCTAGCTCAAAGCTTTTTG	AATTCAAAAAGCTTTGAGCTAGGCTAGAGATCTCGAGATCTCTAGCCTAGCTCAAAGCG
Lv‐sh2‐CERCAM	GATCCGGCCTAGAAATGGCCTCAAATCTCGAGATTTGAGGCCATTTCTAGGCCTTTTTG	AATTCAAAAAGGCCTAGAAATGGCCTCAAATCTCGAGATTTGAGGCCATTTCTAGGCCG

### 2.3 Immunoblotting

RIPA buffer (Catalog number: P0013C; Beyotime) and protease inhibitors were used to obtain total protein from either target tissues or transfected cells or untransfected target cells. The protein concentration was established by the BCA quantitative method (Catalog number: P0012, Beyotime). Then the protein samples were loaded into SDS‐PAGE (8%–15%) for separation and transferred to PVDF membrane. By incubating the membrane with a 5% milk blocking solution for 1 h, non‐specific binding prevention could be achieved. Then the membrane was incubated with anti‐CERCAM (1:500, 16411–1‐AP; Proteintech), anti‐PCNA (1:2000, 10205–2‐AP, Proteintech), anti‐Vimentin (1:2000, 10366–1‐AP, Proteintech), anti‐Twist (1:500, CSB‐PA025358LA01HU; Cusabio), anti‐N‐cadherin (1:2000, 22018–1‐AP, Proteintech), anti‐E‐cadherin (1:5000, 20874–1‐AP, Proteintech), anti‐Akt (Y409094, Abm), anti‐p‐Akt (1:500, Y011054, Phospho‐ser473; Abm), or anti‐β‐actin (1:5000, 60008–1‐Ig, Proteintech) at 4°C for 24 h, and then incubated with the matching secondary antibody at 37°C for 1 h. Enhanced chemiluminescence (ECL) reagents and Automatic chemiluminescence imaging system (Tanon 5200) were used to visualize proteins. Experiments were repeated for 3 times.

### 2.4 Immunohistochemical staining (IHC staining)

Fixed tissues were dehydrated with ethanol and transparent with xylene, finally embedded in paraffin. The tissues were cut into cross‐section of a thickness of 5 μm. Following dewaxing and rehydration, the sections were incubated with a solution of 3% solution of hydrogen peroxide for 10 min to block endogenous peroxidase activity. Then, sections were incubated with a blocking solution (Catalog number: AR1010) for 30 min. Subsequently, tissue sections were incubated with anti‐CERCAM (16411–1‐AP, Proteintech) or anti‐PCNA (10205–2‐AP, Proteintech) overnight at 4°C in a moist chamber and then incubated with SV hypersensitive two‐step kit (Catalog number: SV0004, Boster). The sections were then stained with a DAB chromogenic kit and the sections were then sent for microscope analysis.

### 2.5 Cell lines and cell culture

Ureteral epithelial immortalized cell line, SV‐HUC‐1 (CRL‐9520™), was obtained from ATCC and cultured in F‐12 K Medium (Product Code. 11580556; Gibco) and supplemented with 10% FBS (Catalog number: 10437028; Gibco). This cell line was used as a non‐cancerous control in this study.^16^ Five bladder cancer cell lines, T24 (HTB‐4™),^17^ 5637 (HTB‐9™),^18^ RT4 (HTB‐2),^17^ SW780 (CRL‐2169™),^19^ and J82 (HTB‐1™)^20^ were obtained from ATCC. T24 cells and RT4 cells were then cultured in McCoy's 5a Medium Modified (Catalog number: 16600082, Gibco) supplemented with 10% FBS (Gibco). 5637 cells were cultured in RPMI‐1640 medium (Catalog number: 11875093, Gibco) supplemented with 10% FBS (Gibco). SW780 cells were cultured in Leibovitz's L‐15 Medium (Catalog number: 30–2008, ATCC) and supplemented with 10% FBS (Gibco). J82 cells were cultured in Eagle's Minimum Essential Medium (Gibco) supplemented with 10% FBS (Gibco). All cells were cultured at 37°C in 5% CO_2_.

### 2.6 Lentivirus infection

Lentivirus encoding small interfering RNA targeting CERCAM or lentivirus containing CERCAM‐overexpressing plasmids were generated by GeneChem (Lv‐sh1‐CERCAM/ Lv‐sh2‐CERCAM); Lv‐sh‐NC was used as a negative control. Target cells were plated in 12‐well plates (1 × 10^5^ cells/well), transduced with 5 MOI lentiviral particles using a solution of 8 µg/ml hexadimethrine bromide (Catalog number: H9268, Sigma‐Aldrich), and incubated at 37°C with 5% CO_2_. The overexpression or silencing of CERCAM in stable cells was confirmed by qPCR.

### 2.7 MTT assay for cell viability

The MTT method was performed to assess cell viability.^21^ In general, 1 × 10^5^ chondrocytes were seeded in each well of a 96‐well plate. The MTT solution (10 μl) was added to each well, and the chondrocytes were incubated at 37°C for 4 h. Then, DMSO (150 μl) was added to each well, and the culture plate was placed on a shaker at low speed for 10 min. Finally, the optical density (OD) value was measured at 490 nm with a microplate reader.

### 2.8 EdU assay for DNA synthesis

The target cells were transfected for 48 h and incubated with EdU for 2 h. Following that, the cells were fixed with 4% formaldehyde, washed with PBS, and permeated with a P0097 solution. After Apollo staining and DAPI staining, the number of cells stained with EdU was counted under fluorescence microscopy, and relevant pictures were taken.

### 2.9 Transwell for invasion

For cell invasion using Transwell assay, target cells were planted in the upper chamber insert and coated with Matrix gel (Catalog number: 354234, BD Biosciences) in a serum‐free medium containing 1% FBS. Medium containing 10% FBS was then added to the lower chamber. After an incubation period of 24 h at 37°C, the cells that remained on the upper chamber or upper surface of the membrane were skimmed off. The cells that stuck to the membrane under the insert were stained with DAPI and underwent microscope analysis.

### 2.10 Subcutaneous implant model in nude mice

Male BALB/c nude mice (aged 5–8 weeks old) were purchased from Hunan SJA Laboratory Animal Co., Ltd and utilized for subcutaneous implant model construction. T24 and 5637 cells were then infected with Lv‐sh‐NC or Lv‐sh‐CERCAM and then injected into the nude mice. Each batch of 5 × 10^5^ cells was suspended in 100 μl of serum‐free DMEM, mixed with 100 μl of 10% matrix gel (BD), and injected subcutaneously into the right armpit of each mouse. The tumor size was measured at intervals of 7 days from day 7 following injection. On day 28 after injection (4 weeks after injection), all the mice were anesthetized and killed. Tumor volume, tumor weight, and the protein levels of PCNA and CERCAM in tumor tissues were examined and recorded. All the procedures involved in animal experimentation and handling were carried out in accordance with relevant guidelines and regulations and the approval of the Ethics Committee of Xiangya Hospital of Central South University.

### 2.11 Statistics analysis

Data were expressed as means ± SD of at least three independent experiments. The survival curve was analyzed by CoxPH regression model. The difference of CERCAM expression in TCGA‐BLCA and GEO dataset was analyzed by Kruskal‐Wallis test (more than 2 groups) or *t*‐test (2 groups). The difference between the two groups was examined by *t*‐test. The difference among more than two groups was analyzed by one‐way ANOVA followed by Tukey's multiple comparison test using the Graphpad Prim software. The level of significance was based on the probability of *p* < 0.05, *p *< 0.01.

## 3 RESULTS

### 3.1 CERCAM is markedly related to bladder cancer patients’ survival

Firstly, online data mining was performed based on the data obtained from TCGA‐BLCA. A total of 426 bladder cancer samples from the TCGA‐BLCA data collection was analyzed using a Cox proportional hazards regression model to screen for cell adhesion molecules (CAMs) associated with the survival of patients suffering from bladder cancer, and 8 genes were found to be related to bladder cancer patients' overall survival (|hazard ratio| > 1, *p* < 0.05; Table S2). Among them, CERCAM yielded the highest log (Hazard ratio) value and the lowest p‐value; therefore, further analysis on CERCAM was conducted. As depicted in Figure S1A,B, by using a CoxPH multifactor analysis, CERCAM was closely related to recurrence‐free survival (RFS; hazard ratio = 0.451, *p* = 0.032) and the overall survival (OS; hazard ratio = 0.281, *p* = 0.00013) in patients with bladder cancer. Besides, CERCAM expression increased with the progression of the American Joint Committee on Cancer staging (AJCC staging, namely pathological stage) and TNM staging (Figure S1C,F).

Several datasets from the Gene Expression Omnibus (GEO) also displayed similar results. According to GSE31684, CERCAM was remarkably associated with the RFS (hazard ratio = 0.293, *p* = 0.00026; Figure S2A) and the OS (hazard ratio = 0.337, *p* = 0.0012; Figure S2B), respectively, in patients ailing from bladder cancer. According to GSE31684, CERCAM expression generated a remarkably high level of expression in metastatic (Figure S2C), advanced pre‐operation clinical stages (Figure S2D) tissue samples. According to GSE120736, CERCAM expression was higher within the muscle‐invasive subtype than that in non‐muscle‐invasive subtype (Figure S2E) and in advanced stages compared with that of early stages (Figure S2F). These data indicate that CERCAM can worsen prognosis rates in bladder cancer patients; CERCAM expression is higher in advanced stage samples and invasive samples.

### 3.2 Tissue and cellular expression of CERCAM

**FIGURE 1:**
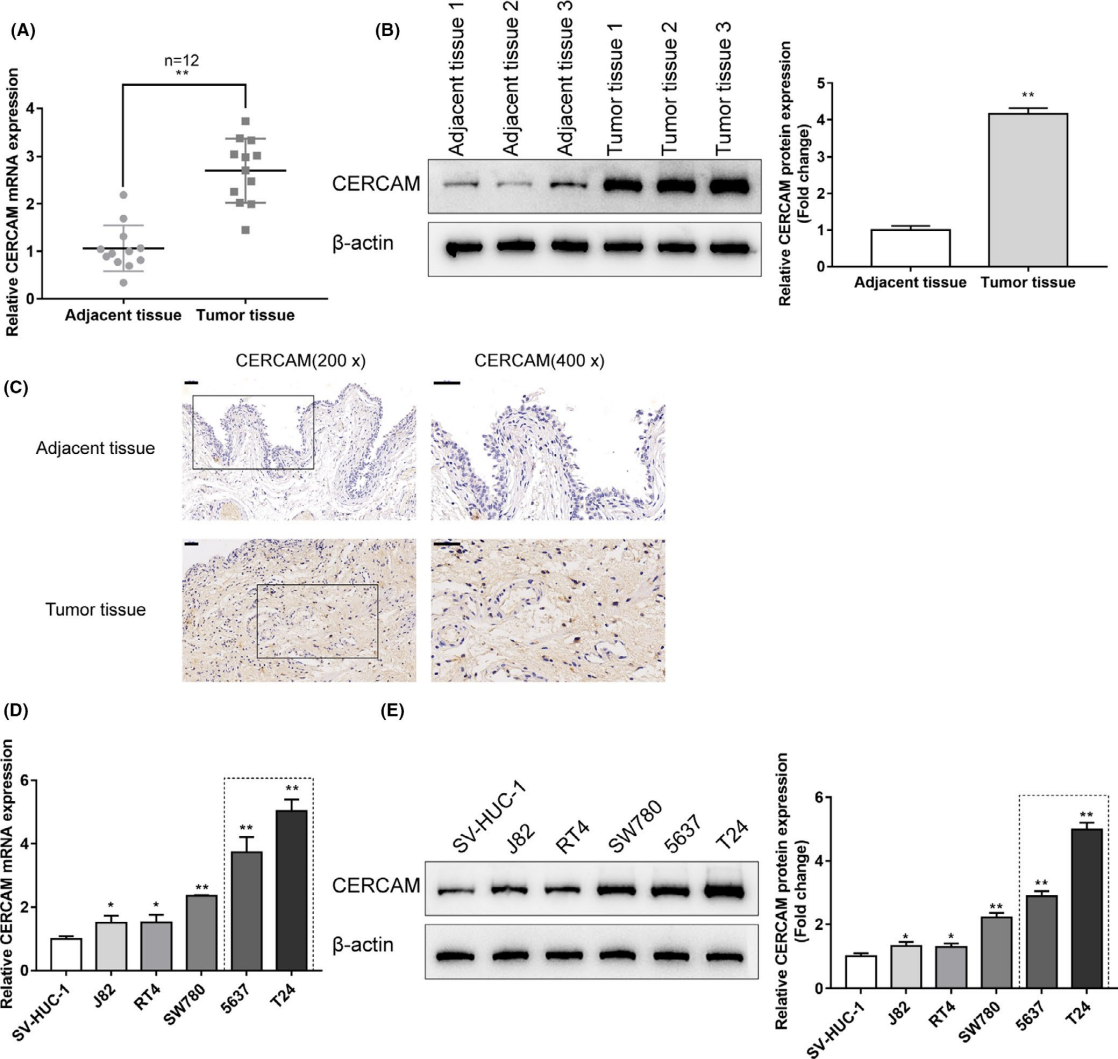
Tissue and cellular expression of CERCAM (A) Twelve paired bladder cancer tissues and adjacent non‐cancerous tissues were harvested, diagnosed, and assessed for the expression of CERCAM mRNA in tissue samples using qRT‐PCR. CERCAM upregulation in tumor tissues was observed. *N* = 12, *t*‐test. (B) The protein levels of CERCAM in tissue samples were examined using Immunoblotting and an increase in CERCAM protein level was observed in tumor tissues. *N* = 3, *t*‐test. (C) The protein content and distribution of CERCAM in the tissue samples were examined using an immunohistochemical (IHC) staining. CERCAM‐positive area was increased in tumor tissues. (D) The expression of CERCAM mRNA in a normal ureteral epithelial immortalized cell line, SV‐HUC‐1, and five bladder cancer cell lines, T24, 5637, RT4, SW780, and J82, was determined using qRT‐PCR. CERCAM upregulation in cancer cells was observed. *N* = 3, One‐way ANOVA test. F value is 91.19. (E) The protein levels of CERCAM were examined in SV‐HUC‐1, T24, 5637, RT4, SW780, and J82 cells using Immunoblotting. An increase in CERCAM protein level was observed in cancer cells. *N* = 3, One‐way ANOVA test. F value is 320.8. *p < 0.05, ** *p* < 0.01

To further confirm CERCAM expression within bladder carcinoma, 12 paired bladder carcinoma tissue samples and adjacent normal control tissue samples were collected and examined for CERCAM expression and protein contents within tissue samples. Figure 1A,B shows that CERCAM mRNA and protein expression showed to be dramatically higher within bladder carcinoma tissue samples as compared to the adjacent non‐cancerous tissue samples. CERCAM protein content and distribution were further proved through IHC staining (Figure 1C). As for the cellular expression, in comparison with that in a normal ureteral epithelial immortalized cell line, SV‐HUC‐1, CERCAM expression and protein levels were found to be significantly higher in five bladder carcinoma cell lines, T24, 5637, RT4, SW780, and J82 (Figure 1D,E); among the five bladder carcinoma cell lines, CERCAM expression and protein levels were higher in T24 and 5637 cells (Figure 1D,E). Consequently, T24 and 5637 cells were chosen for further investigation.

### 3.3 Specific effects of CERCAM overexpression and silencing on bladder cancer cell phenotypes

**FIGURE 2:**
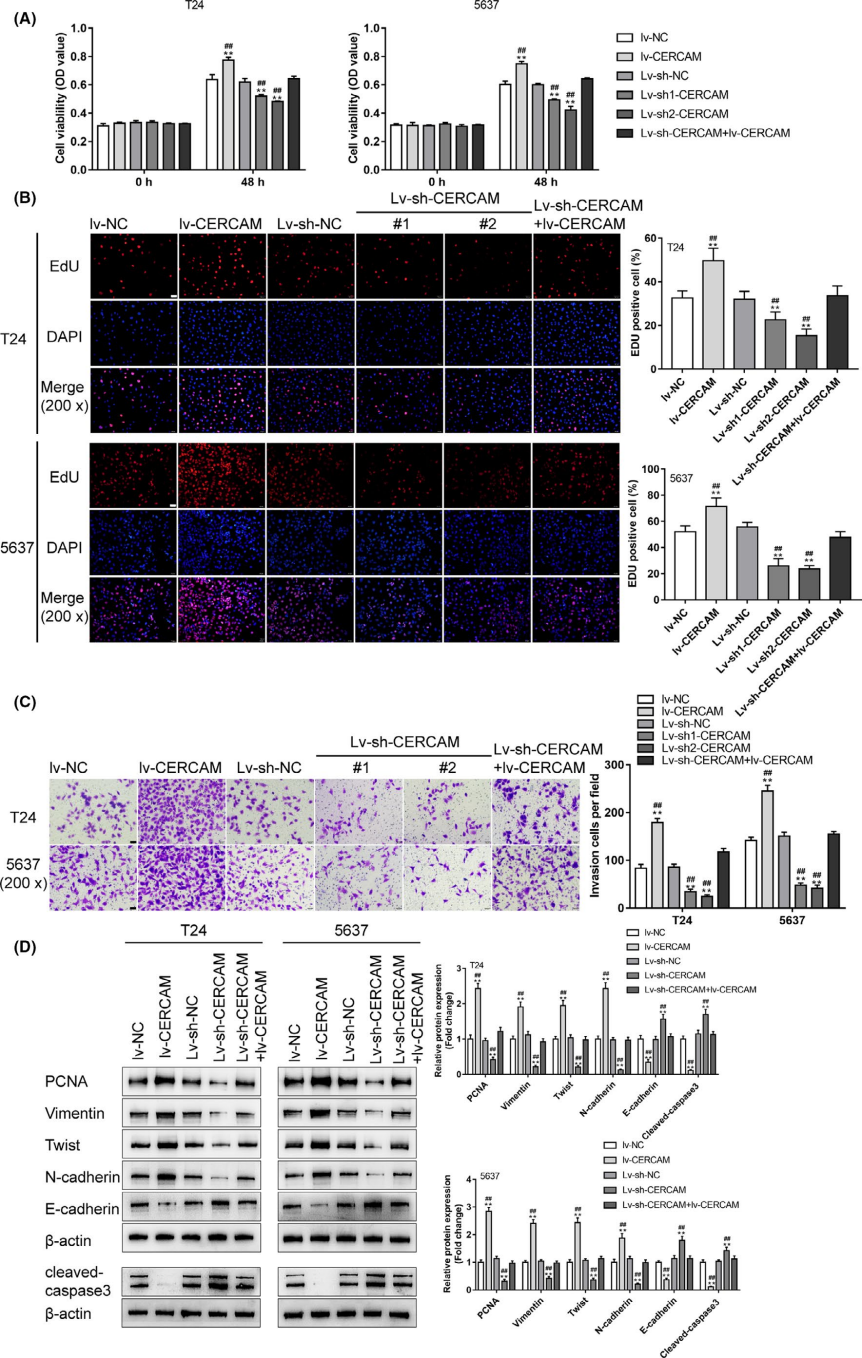
Specific effects of CERCAM overexpression and silencing on bladder cancer cell phenotypes T24 and 5637 cells were infected with lv‐NC/lv‐CERCAM or lv‐sh‐NC/lv‐sh1‐CERCAM/lv‐sh2‐CERCAM for conducting CERCAM overexpression or silencing. Subsequently, T24 and 5637 cells were infected with lv‐NC/lv‐CERCAM or lv‐sh‐NC/lv‐sh1‐CERCAM/ lv‐sh1‐CERCAM and examined for cell viability through an MTT assay (A), *n* = 5, One‐way ANOVA test, F value is 118.7 (T24 cells) and 237.5 (5637 cells). DNA synthesis was determined through an EdU assay (B), *n* = 3, One‐way ANOVA test, F value is 24.85 (T24 cells) and 44.5 (5637 cells). Cell invasion ability was determined by a Transwell assay (C), *n* = 3, F value is 211.5 (T24 cells) and 289.2 (5637 cells). The protein levels of PCNA, Vimentin, Twist, N‐cadherin, E‐cadherin, and cleaved‐caspase3 by immunoblotting (D), *n* = 3, One‐way ANOVA test. PCNA: F value is 160.7 (T24 cells) and 284.3 (5637 cells). Vimentin: F value is 128.0 (T24 cells) and 245.4 (5637 cells). Twist: F value is 130.2 (T24 cells) and 166.2 (5637 cells). N‐cadherin: F value is 223.1 (T24 cells) and 91.32 (5637 cells). E‐cadherin: F value is 62.51 (T24 cells) and 86.94 (5637 cells). cleaved‐caspase3: F value is 109.0 (T24 cells) and 93.42 (5637 cells). The malignant behaviors of cancer cells were promoted by CERCAM overexpression but inhibited by CERCAM knockdown. **p < 0.01, compared to lv‐NC. #*p* < 0.05, ##*p* < 0.01, compared to lv‐sh‐NC

T24 and 5637 cells were infected with lv‐NC/lv‐CERCAM or lv‐sh‐NC/lv‐sh1‐CERCAM/lv‐sh2‐CERCAM for conducting CERCAM overexpression or silencing. The lentivirus infection efficiency is shown in Figure S3A. Around 90% bladder cancer cells were successfully infected. qRT‐PCR (Figure S3B,C) and immunoblotting (Figure S3D,E) were employed to confirm CERCAM expression. Lv‐sh2‐CERCAM was chosen for further experiments due to its more efficient inhibitory properties (Figure S3C,E). Then, T24 and 5637 cells were infected with lv‐NC/lv‐CERCAM or lv‐sh‐NC/lv‐sh1‐CERCAM/lv‐sh2‐CERCAM and assessed for bladder cancer cell viability and DNA synthesis ability. Figure 2A‐B shows that CERCAM overexpression was significantly promoted, whereas CERCAM silencing suppressed the viability and DNA synthesis ability of bladder cancer cells compared with that within the lv‐NC or lv‐sh‐NC group. Moreover, as shown in Figure 2C, CERCAM overexpression significantly promoted, whereas CERCAM silencing by lv‐sh1‐CERCAM and lv‐sh2‐CERCAM suppressed bladder cancer cell invasion compared to that in the lv‐NC or lv‐sh‐NC group. As for the protein levels of the proliferating marker PCNA and migratory markers: Vimentin, Twist, N‐cadherin, E‐cadherin, and cleaved‐caspase3, CERCAM overexpression significantly increased levels of PCNA, Vimentin, Twist, and N‐cadherin proteins but decreased the levels of E‐cadherin and cleaved‐caspase3, whereas CERCAM silencing exhibited opposite effects on these markers (Figure 2D). More importantly, in the T24 and 5637 cells that were co‐transduced with Lv‐sh‐CERCAM and lv‐CERCAM, the tumor‐suppressive effects of CERCAM knockdown on cell viability, DNA synthesis, cell invasion, and related markers were partially impaired by CERCAM overexpression (Figure 2A,D).

To re‐iterate the tumor‐promotive effects of CERCAM overexpression, CERCAM was overexpressed in a non‐cancerous cell line, SV‐HUC‐1. In SV‐HUC‐1 cells, CERCAM overexpression significantly promoted cell viability (Figure S4A) and cell invasion (Figure S4B).

### 3.4 In vivo effects of CERCAM silencing on subcutaneously implanted tumor growth

**FIGURE 3:**
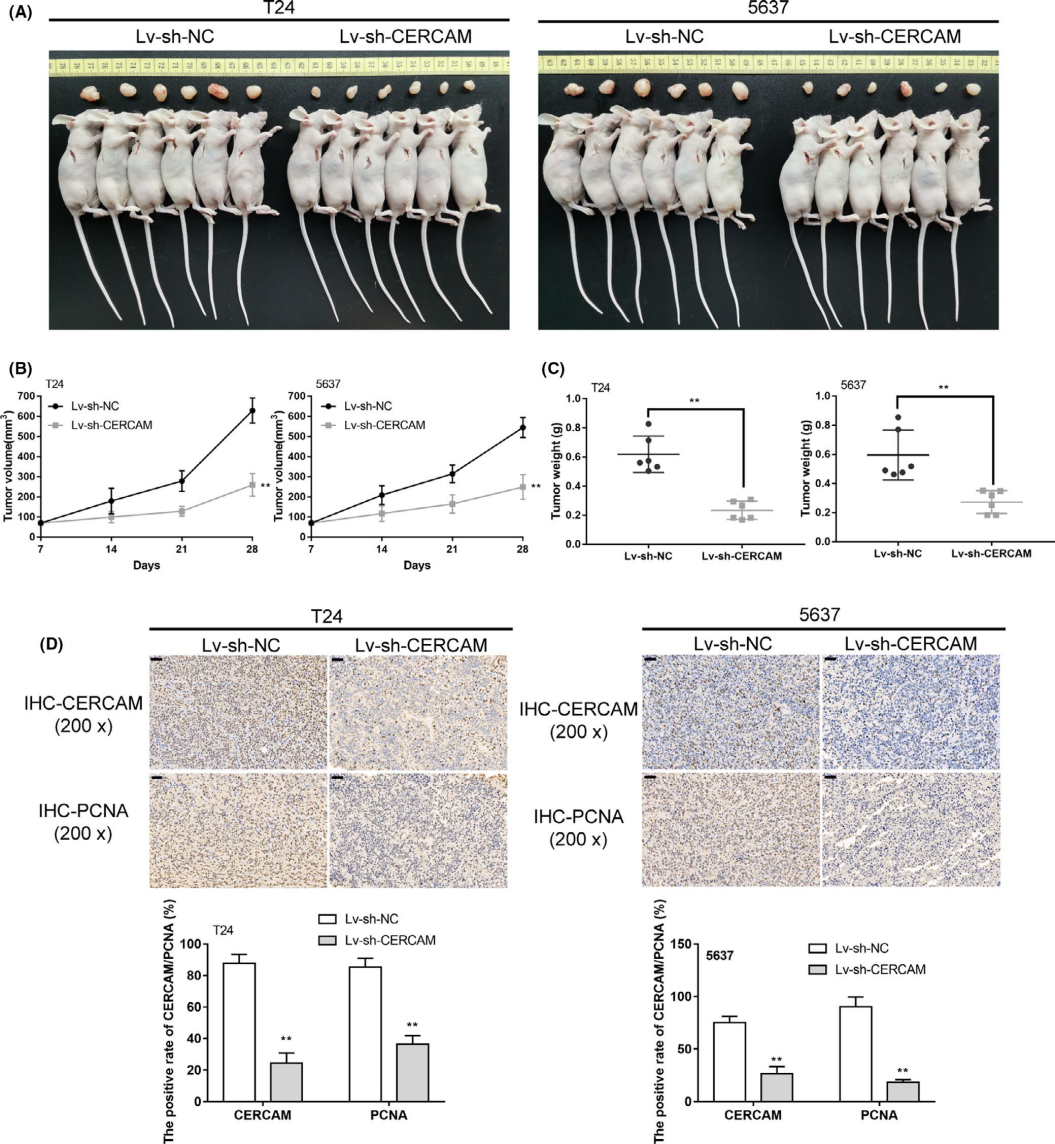
In vivo effects of CERCAM silencing on subcutaneously implanted tumor growth (A) lv‐sh‐NC or lv‐sh‐CERCAM infected T24 and 5637 cells were injected to the right axilla of the nude mouse as described. (B) Tumor size was measured every 7 days starting on day 7 of the injection. On day 28 of the injection, mice were anesthetized and killed. Tumor weight was measured (C). *n* = 6, *t*‐test. The protein levels of PCNA and CERCAM in tumor tissues were examined using an immunohistochemical (IHC) staining (D). *n* = 3, *t*‐test. The tumor growth in the mice model was inhibited by CERCAM knockdown. **p < 0.01, ##*p *< 0.01

To further confirm the in vitro findings, a subcutaneous implant tumor model was conducted in nude mice by injecting T24 or 5637 cells infected with lv‐sh‐NC or lv‐sh‐CERCAM as described (Figure 3A). The tumor size progression was measured and recorded in cycles of 7 days starting at day 7 following injection; as shown in Figure 3B, in vivo CERCAM silencing efficiently reduced the tumor volume. On day 28 of the injection, the mice were anesthetized and killed; in vivo CERCAM silencing significantly decreased the tumor weight (Figure 3C). Also, in vivo CERCAM silencing decreased the protein levels of PCNA and CERCAM in tumor tissues compared with those in the lv‐sh‐NC group (Figure 3D). It was to be concluded that in vivo CERCAM silencing would suppress the growth of subcutaneously implanted tumors.

### 3.5 The PI3K/AKT pathway participates in the effects of CERCAM on bladder cancer cell proliferation

**FIGURE 4:**
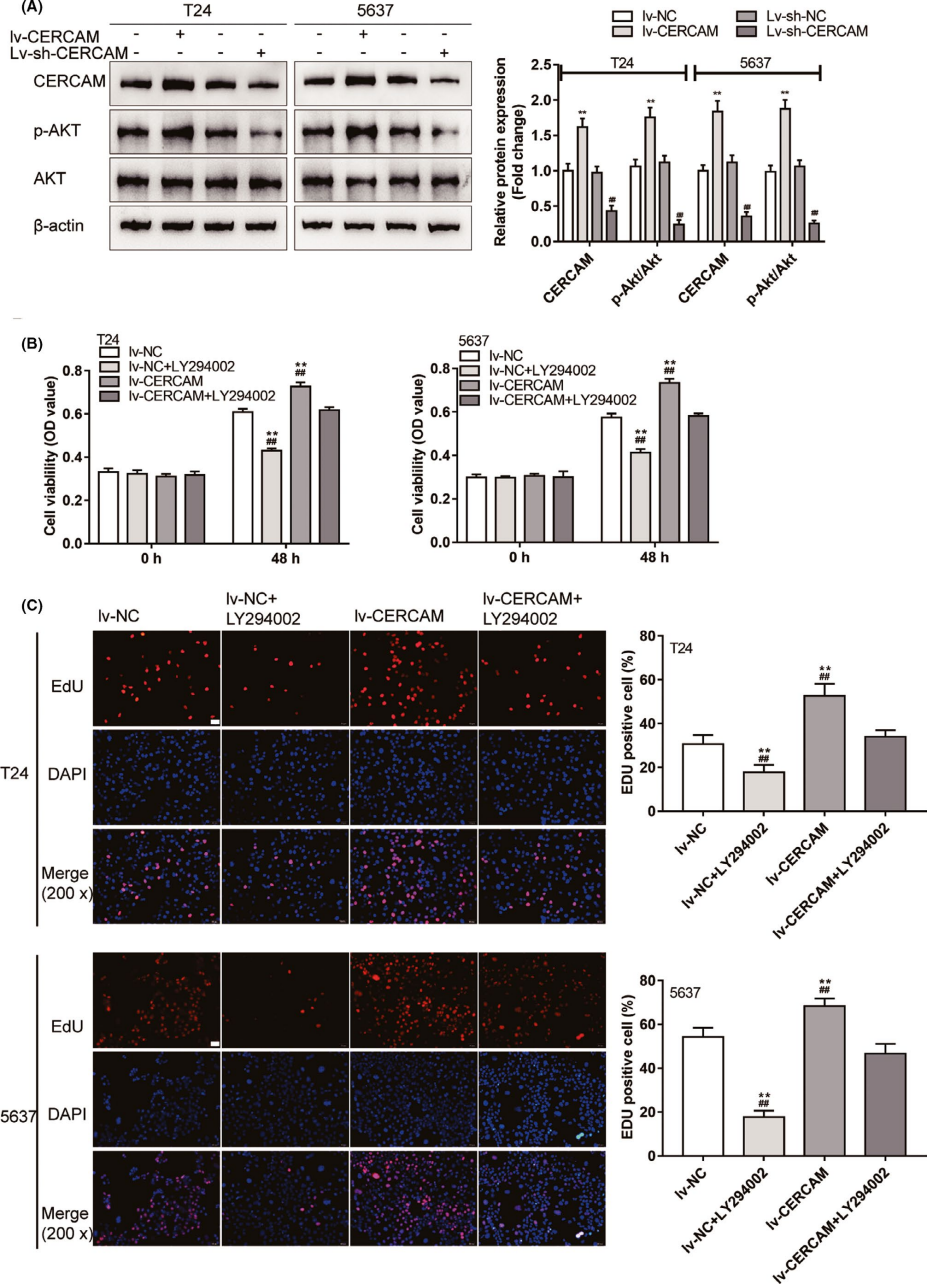
The PI3K/AKT signaling is involved in the effects of CERCAM on bladder cancer cell proliferation (A) T24 and 5637 cells were infected with lv‐NC/lv‐CERCAM or lv‐sh‐NC/lv‐sh‐CERCAM and examined for the protein levels of p‐AKT, and AKT by Immunoblotting. CERCAM overexpression promoted Akt phosphorylation. *n* = 3, One‐way ANOVA test. CERCAM: F value is 58.31 (T24 cells) and 79.52 (5637 cells). p‐Akt/Akt: F value is 87.32 (T24 cells) and 115.7 (5637 cells). Then, T24 and 5637 cells were infected with lv‐NC/lv‐CERCAM with or without PI3K inhibitor LY294002 and examined for cell viability by an MTT assay (B). *n* = 5, One‐way ANOVA test. F value is 260.4 (T24 cells) and 254.8 (5637 cells). DNA synthesis was determined by an EdU assay (C). *n* = 3, One‐way ANOVA test. F value is 36.29 (T24 cells) and 92.31 (5637 cells). The promotive effects of CERCAM overexpression on cancer cell proliferation were partially abated by LY294002. **p < 0.01, compared to lv‐NC. #*p *< 0.05, ##*p *< 0.01, compared to lv‐sh‐NC or lv‐CERCAM+LY294002

It has been reported that alterations to the PI3K‐Akt signaling pathway are commonly seen in human cancers, including bladder cancer.^14^ Considering its effect on the modulation of cell growth, survival, and metastasis, the involvement of the PI3K/AKT signaling in CERCAM functions on bladder carcinoma cell proliferation was then investigated. First, the alterations of AKT phosphorylation in CERCAM‐overexpressing or CERCAM‐silenced T24 and 5637 cells were examined, as shown in Figure 4A, CERCAM overexpression significantly increased the CERCAM protein level and the ratio of p‐AKT/AKT, whereas CERCAM silencing triggered opposite effects. Then, T24 and 5637 cells were infected with lv‐NC/lv‐CERCAM with or without PI3K inhibitor LY294002 and examined for cell viability and DNA synthesis. CERCAM overexpression significantly promoted, whereas LY294002 treatment inhibited cell viability and DNA synthesis; the promotive effects of CERCAM overexpression were partially attenuated with LY294002 treatment (Figure 4B,C).

### 3.6 The PI3K/AKT pathway participates in the effects of CERCAM on bladder cancer cell invasion

**FIGURE 5:**
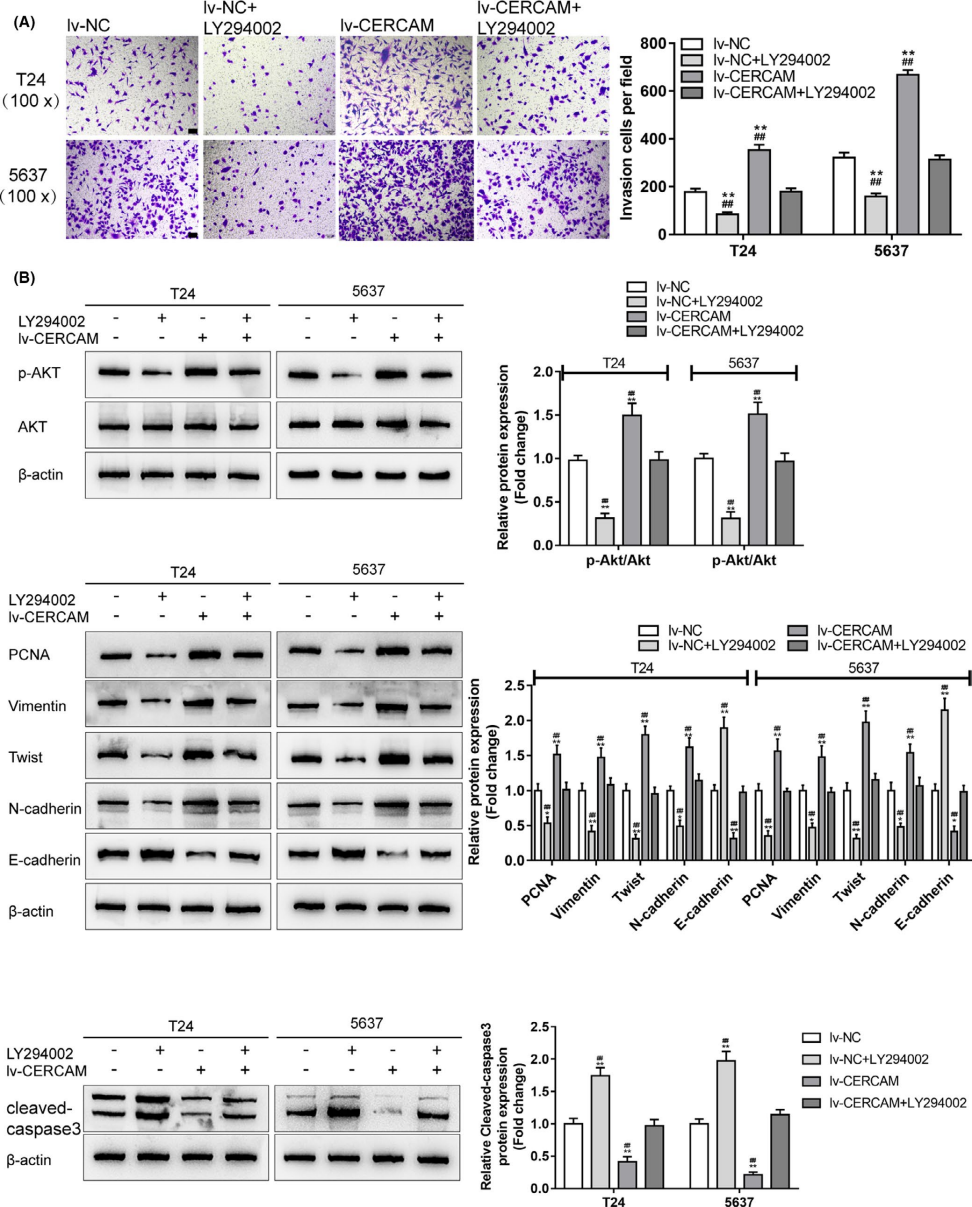
The PI3K/AKT signaling is involved in the effects of CERCAM on bladder cancer cell invasion T24 and 5637 cells were infected with lv‐NC/lv‐CERCAM with or without PI3K inhibitor LY294002 and examined for cell invasion by Transwell assay (A). *n* = 3, One‐way ANOVA test. F value is 154.3 (T24 cells) and 414.3 (5637 cells). The protein levels of p‐AKT, AKT, PCNA, Vimentin, Twist, N‐cadherin, E‐cadherin, and cleaved‐caspase3 were determined by immunoblotting (B). *n* = 3, One‐way ANOVA test. PCNA: F value is 42.19 (T24 cells) and 61.47 (5637 cells). Vimentin: F value is 49.97 (T24 cells) and 49.24 (5637 cells). Twist: F value is 121.3 (T24 cells) and 111.7 (5637 cells). N‐cadherin: F value is 67.02 (T24 cells) and 48.19 (5637 cells). E‐cadherin: F value is 111.4 (T24 cells) and 124.5 (5637 cells). cleaved‐caspase3: F value is 92.49 (T24 cells) and 176.9 (5637 cells). The effects of CERCAM overexpression on these factors were partially attenuated by LY294002. **p < 0.01, compared to lv‐NC. ##*p *< 0.01, compared to lv‐CERCAM+LY294002

Next, the dynamic effects of CERCAM overexpression and LY294002 upon T24 and 5637 cell invasion and migratory markers were further confirmed. Similarly, CERCAM overexpression significantly promoted, whereas LY294002 treatment inhibited cell invasion; the promotive effects of CERCAM overexpression were partially attenuated under LY294002 treatment (Figure 5A). As for the alterations in PI3K/AKT signaling, CERCAM overexpression increased, whereas LY294002 treatment decreased p‐AKT/AKT; the effects of CERCAM overexpression were attenuated under LY294002 treatment (Figure 5B). As for the migratory markers, CERCAM overexpression increased the protein levels of PCNA, Vimentin, Twist, N‐cadherin, and decreased E‐cadherin and cleaved‐caspase3, whereas CERCAM silencing had opposite effects on these proteins; the impacts of CERCAM overexpression were attenuated under LY294002 treatment (Figure 5B).

## 4 DISCUSSION

**FIGURE 6:**
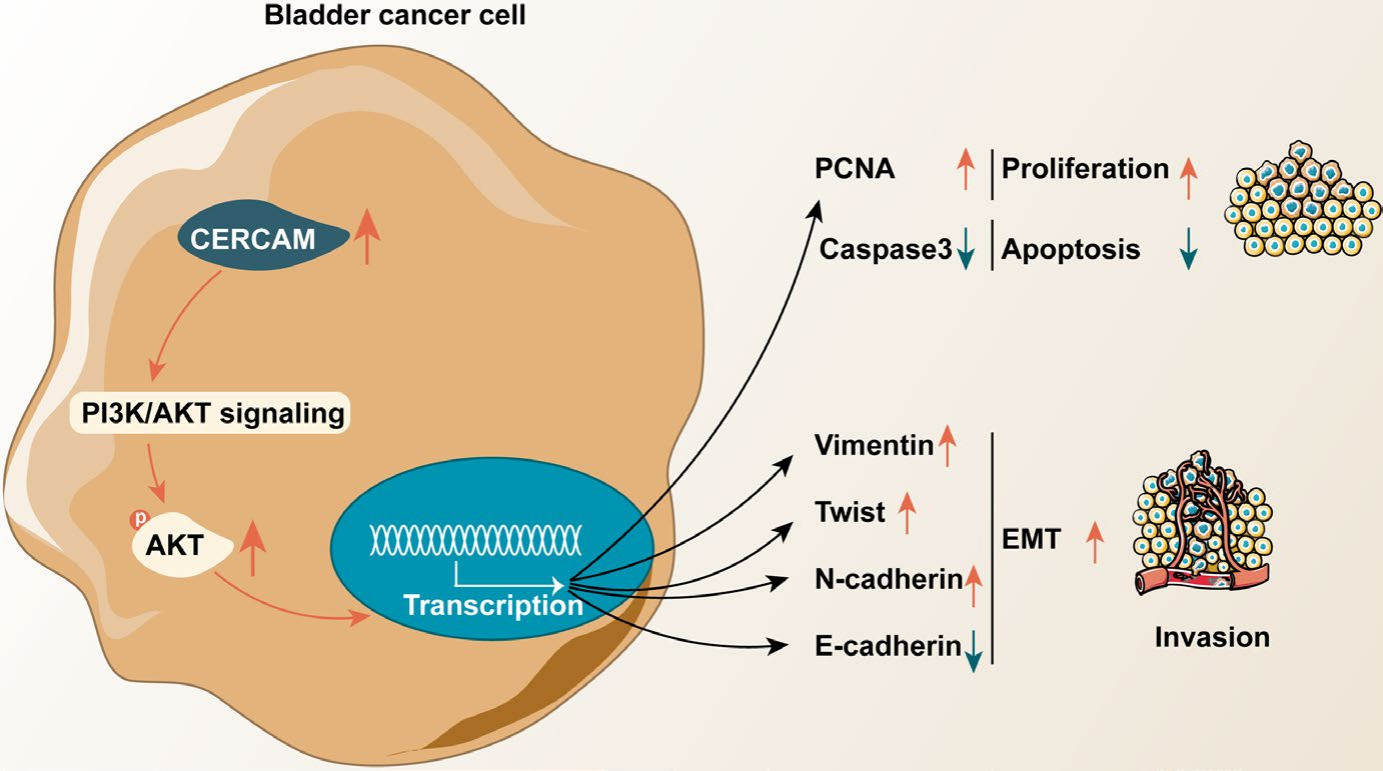
A schematic diagram showing the *in vitro* and in vivo role of CERCAM in bladder cancer and underlying mechanism *In vitro*, CERCAM overexpression not only significantly promotes bladder cancer cell viability, DNA synthesis, and cell invasion but also alters E‐cadherin and N‐cadherin expression pattern; in vivo, CERCAM silencing suppressed tumor growth in nude mice. The PI3K/AKT signaling is thought to participate in the functions of CERCAM in bladder cancer

The study predicted that a higher CERCAM levels is associated with lower rates of patient survival; CERCAM expression is higher in advanced stage samples and invasive samples. In collected clinical tissue samples, the expression of CERCAM was shown to be increased within bladder carcinoma tissues rather than in non‐cancerous tissue controls. In bladder cancer cell lines, CERCAM was also upregulated compared with that in normal cells. In vitro, CERCAM overexpression significantly promoted bladder‐cancer cell viability, DNA synthesis, and cell invasion, whereas CERCAM silencing suppressedthe viability of bladder cancer cell, DNA synthesis, and cell invasion compared with that in the lv‐NC or lv‐sh‐NC group. CERCAM overexpression significantly increased the levels of PCNA, Vimentin, Twist, and N‐cadherin proteins but decreased E‐cadherin and cleaved‐caspase3, whereas CERCAM silencing had opposite effects on these markers. In vivo, CERCAM silencing would suppress the growth of subcutaneously implanted tumors. CERCAM altered the phosphorylation of AKT. The PI3K inhibitor LY294002 treatment exerted similar effects as did CERCAM on silencing bladder cancer cell behaviors. It partially neutered the promotive roles of CERCAM overexpression in the ability of bladder cancer cells to proliferate and to invade (Figure 6).

The effect of cell adhesion molecules on bladder carcinoma is multifaceted and complex. For example, the cell adhesion molecule 1 (CADM1), which acts as a tumor suppressor, appeared to be under‐expressed within bladder carcinoma tissues. CADM1 protein overexpression suppressed the proliferative ability, decreased the invasive ability, and triggered the apoptosis of tumor cells in vitro.^22^ Another cell adhesion molecule, the carcinoembryonic antigen‐related cell adhesion molecule 1 (CEACAM1), exhibited differential expression within the epithelium of the superficial bladder tumors and the prostate intraepithelial neoplasia of the prostate; however, in both cancers, CEACAM1 overexpression effectively suppressed tumor vascularization.^23^ CEACAM1 knockdown induced a notable surge in the vascular endothelial growth factors (VEGF)‐C and VEGF‐D. Accordingly, supernatants from the CEACAM1‐overexpressing bladder cancer cell lines inhibited the formation of endovascular and enhance the morphogenesis of VEGF.^24^ Considering the critical effect of cell adhesion molecules on bladder cancer progression, the correlation between cell adhesion molecules and bladder cancer patients' survival was analyzed. Through the use of bioinformatics analysis, CERCAM was found to have exhibited a significant association with bladder cancer patients' RFS and OS.

CERCAM is an adhesion molecule expressed at high levels in brain microvessels that may be used by leukocytes to stretch across the blood‐brain barrier, adjusting white blood cell migratory ability and adhesion.^25^ Although it has been predicted as a downstream target of hsa‐let‐7a, which might contribute to hepatitis, metabolism, and immunity and exert critical effects on play key roles in the pathological physiology of acute‐on‐chronic liver failure,^26^ its specific role in bladder cancer carcinogenesis has never been investigated. According to a series of online datasets, CERCAM expression was higher in advanced stages and invasive tissue samples. Results of the experimental analysis show an abnormal upregulation of CERCAM in tissues and cells of bladder carcinoma. In vitro, CERCAM overexpression markedly enhanced the viability, DNA synthesizing capacity, and invasive ability of bladder carcinoma cells. Notably, CERCAM overexpression remarkably increased N‐cadherin levels but decreased E‐cadherin levels in bladder cancer cells. As mentioned before, E‐cadherin expression was decreased while N‐cadherin expression was elevated, which could potentially enhance cell migration into tissues,^9^, ^10^, ^12^ as well as induce alterations in intracellular signaling to promote cell growth and invasive abilities.^9^, ^12^ These alterations in Vimentin, Twist, N‐cadherin, and E‐cadherin levels further proved and explained the promotive roles of CERCAM overexpression in the ability of bladder carcinoma cells to proliferate and to invade. Consistently, CERCAM silencing suppressed tumor growth in the subcutaneous implant tumor model in vivo, further indicating the underlying effect of CERCAM on bladder cancer.

It has been reported that alterations to the PI3K‐Akt signaling pathway are commonly seen in human cancers, including bladder cancer. Within bladder cancer, PI3K/AKT signaling over‐activation was frequently observed. The PI3K signaling pathway is a crucial target for various classes of compounds that suppress PI3K, AKT, mTORC1, mTORC1 and mTORC2, PI3K, and mTOR.^16^ These agents may induce different therapeutic effects. In this study, enhanced AKT phosphorylation in response to CERCAM overexpression and suppressed AKT phosphorylation in response to CERCAM silencing was observed. CERCAM‐mediated alteration in AKT phosphorylation suggests the potential involvement of the PI3K/AKT signaling in CERCAM functions in bladder cancer. As expected, PI3K inhibitor LY294002 treatment significantly impaired bladder cancer cell viability, DNA synthesis, and cell invasion. More importantly, in CERCAM‐overexpressing bladder cancer cells, LY294002 treatment partially abated the promotive roles of CERCAM overexpression in the malignant behaviors of bladder carcinoma cells.

In conclusion, cell adhesion molecule CERCAM is overexpressed in bladder cancer tissues. In vitro, CERCAM overexpression not only significantly promotes bladder cancer cell viability, DNA synthesis, and cell invasion, but also alters E‐cadherin and N‐cadherin expression pattern; in vivo, CERCAM silencing suppressed tumor growth in nude mice. The PI3K/AKT signaling is suspected of participating in the functions of CERCAM in bladder cancer.

## CONFLICT OF INTEREST

None.

## ETHICS APPROVAL STATEMENT

The clinical sampling was performed with the approval of the Medical Institutional Ethics Committee of Xiangya Hospital, Central South University. All the animal procedures were carried out in accordance with the relevant guidelines and regulations and the approval of the Ethics Committee of Xiangya Hospital of Central South University.

## Supporting information

Fig S1

Fig S2

Fig S3

Fig S4

Table S1

Table S2

## Data Availability

All data generated or analysed during this study are included in this published article.
