# Supplementary figures and images for: The oncogenic role of the cerebral endothelial cell adhesion molecule (CERCAM) in bladder cancer cells in vitro and in vivo
Source: Cancer Med. 2021 Jun 8;10(13):4437–50. doi: 10.1002/cam4.3955 (PMC8267158; doi:10.1002/cam4.3955)

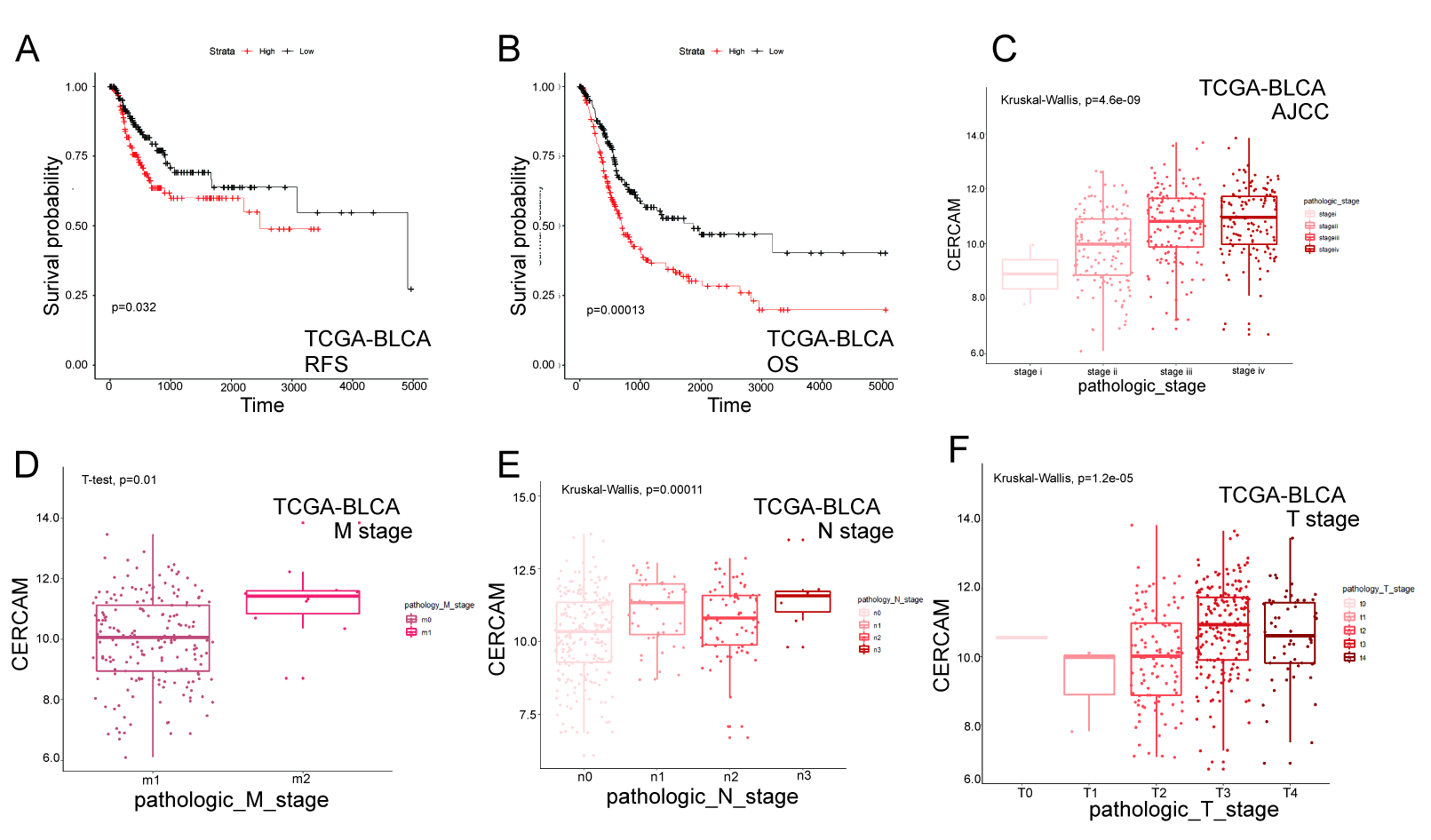

Supplement: Supplementary file 1 — Fig S1 [file CAM4-10-4437-s001.tif]

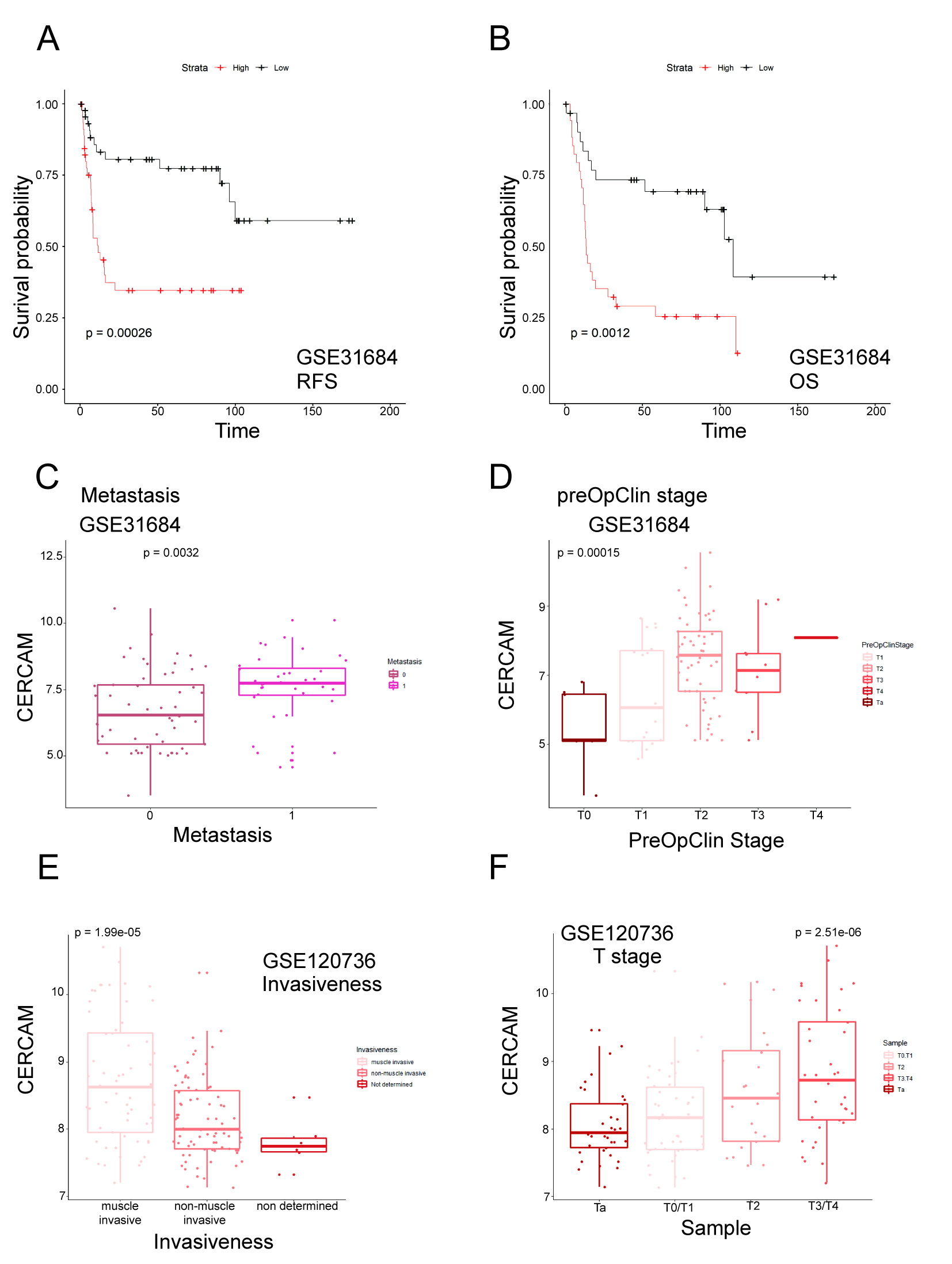

Supplement: Supplementary file 2 — Fig S2 [file CAM4-10-4437-s005.tif]

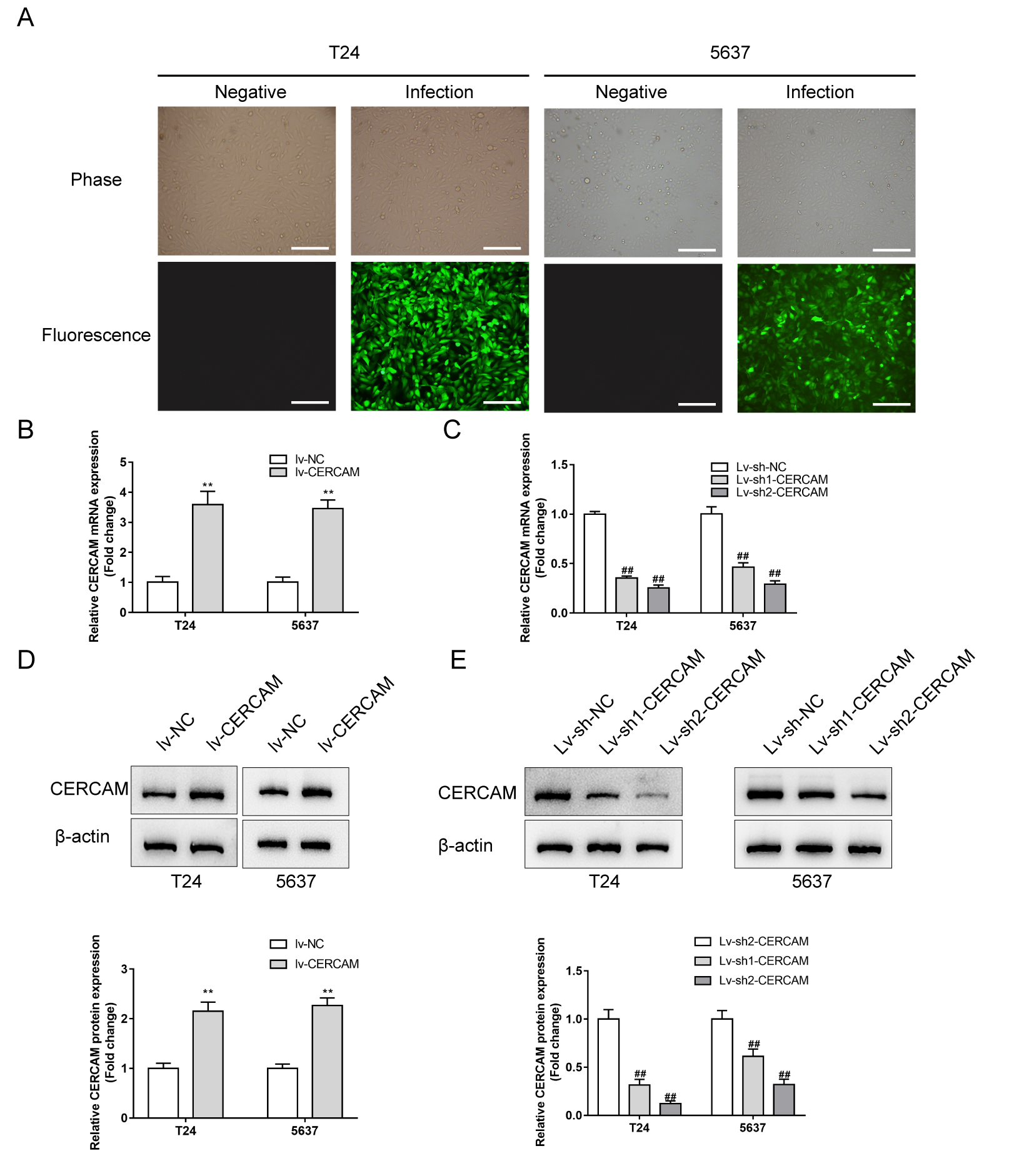

Supplement: Supplementary file 3 — Fig S3 [file CAM4-10-4437-s004.tif]

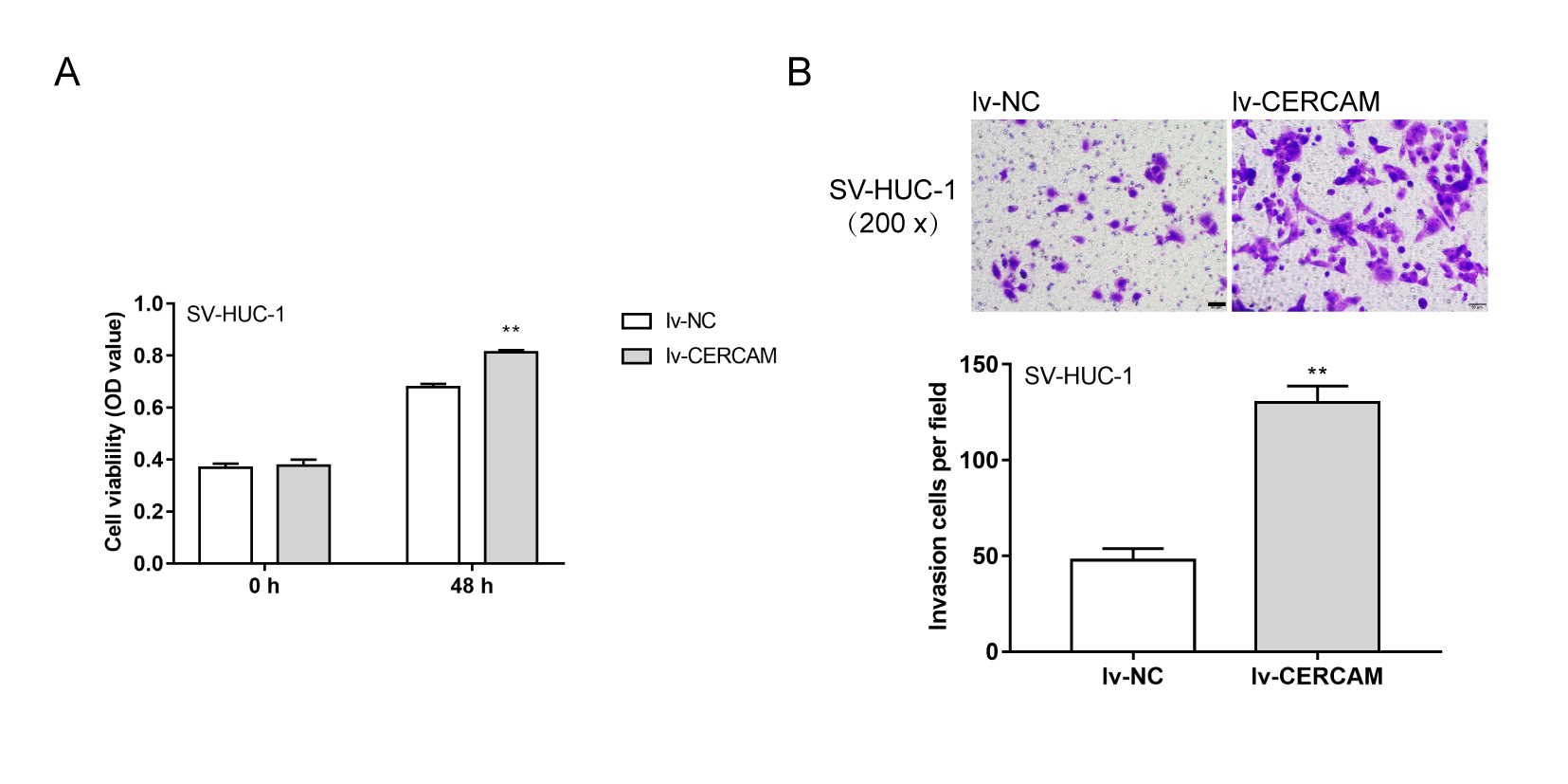

Supplement: Supplementary file 4 — Fig S4 [file CAM4-10-4437-s003.tif]
