# Supplementary material for: The oncogenic role of the cerebral endothelial cell adhesion molecule (CERCAM) in bladder cancer cells in vitro and in vivo
Source: Cancer Med. 2021 Jun 8;10(13):4437–50. doi: 10.1002/cam4.3955 (PMC8267158; doi:10.1002/cam4.3955)
Supplement: Supplementary file 5 — Table S1 [file CAM4-10-4437-s002.docx]

**Table S1. The clinicopathologic characteristics of 12 Bladder Cancer patients**

| **Case number** | **Gender** | **Age at surgery** | **Single/Multiple** | **TNM stage** |
| --- | --- | --- | --- | --- |
| Patient_1 | Female | 30 | Single | T1N0M0 |
| Patient_2 | Male | 65 | Multiple | T2N0M0 |
| Patient_3 | Male | 42 | Multiple | T3N1M1 |
| Patient_4 | Female | 51 | Single | T1N0M0 |
| Patient_5 | Male | 61 | Multiple | T3N2M1 |
| Patient_6 | Female | 65 | Single | T3N2M1 |
| Patient_7 | Male | 42 | Single | T2N0M0 |
| Patient_8 | Male | 67 | Multiple | T3N2M1 |
| Patient_9 | Female | 28 | Single | T1N0M0 |
| Patient_10 | Male | 39 | Multiple | T3N1M1 |
| Patient_11 | Female | 62 | Single | T2N0M0 |
| Patient_12 | Male | 58 | Single | T2N0M0 |
