# Supplementary material for: The oncogenic role of the cerebral endothelial cell adhesion molecule (CERCAM) in bladder cancer cells in vitro and in vivo
Source: Cancer Med. 2021 Jun 8;10(13):4437–50. doi: 10.1002/cam4.3955 (PMC8267158; doi:10.1002/cam4.3955)
Supplement: Supplementary file 6 — Table S2 [file CAM4-10-4437-s006.docx]

**Table S2 Genes associated with the overall survival in patients with bladder cancer**

| Gene/Attribute | Log(Hazard ratio) | P-value | FDR |
| --- | --- | --- | --- |
| CERCAM | 2.81E-01 | 2.71E-06 | 3.46E-03 |
| NCAM1 | 9.53E-02 | 7.53E-04 | 3.04E-02 |
| DSCAM | 1.38E-01 | 9.36E-04 | 3.28E-02 |
| MCAM | 2.05E-01 | 3.56E-03 | 6.04E-02 |
| ICAM5 | 9.97E-02 | 1.08E-02 | 1.05E-01 |
| NCAM2 | 8.13E-02 | 1.18E-02 | 1.11E-01 |
| EPCAM | -1.08E-01 | 1.48E-02 | 1.23E-01 |
| CERCAM1 | -9.72E-02 | 1.69E-02 | 1.31E-01 |
| TCAM1P | -6.34E-02 | 3.26E-02 | 1.84E-01 |
